# Supplementary material for: Loss of ASD-related molecule Cntnap2 affects colonic motility in mice
Source: Front Neurosci. 2023 Nov 9;17:1287057. doi: 10.3389/fnins.2023.1287057 (PMC10665486; doi:10.3389/fnins.2023.1287057)
Supplement: Supplementary file 1 [file Presentation_1.pdf]

frontiers

Supplementary Material

Supplemental Figure 1

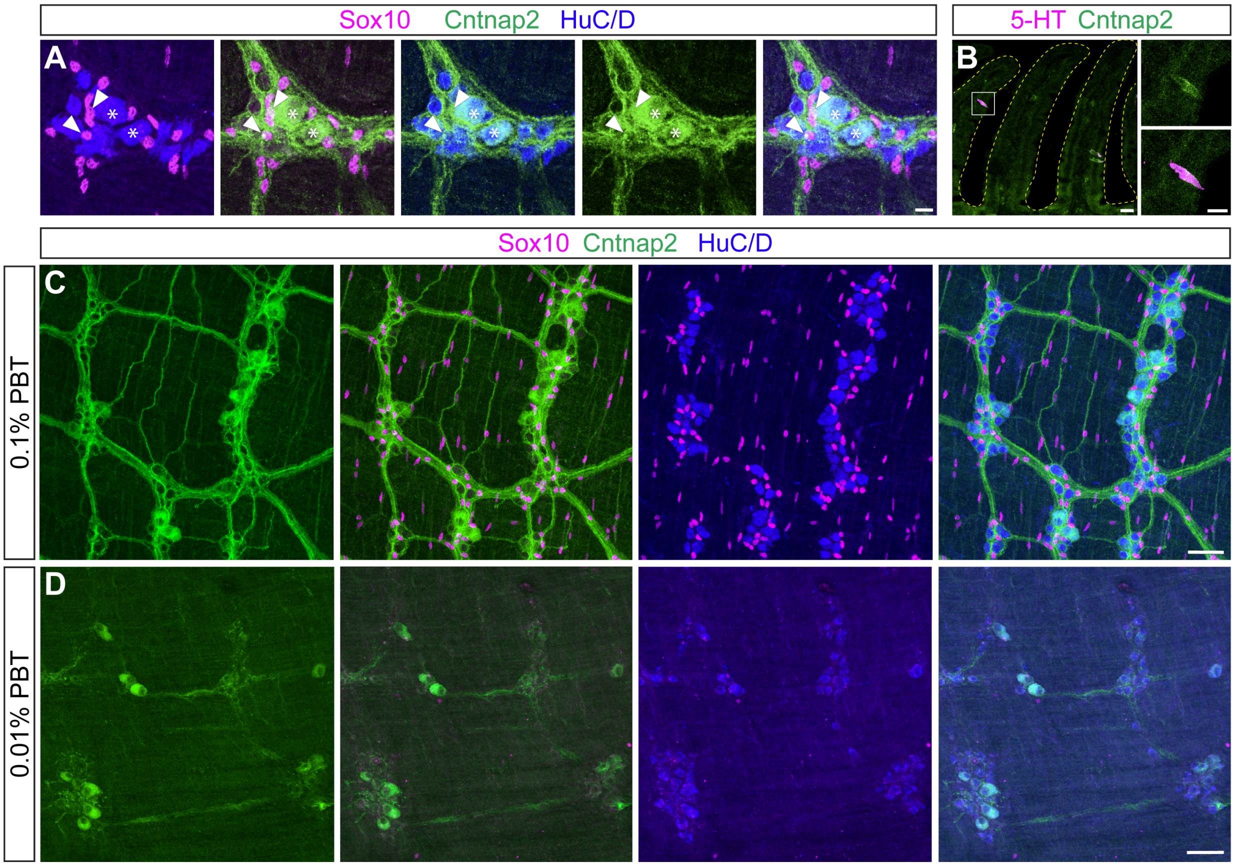

Figure S1. Cntnap2 colocalizes with a subset of progenitor/glial and enteroendocrine cells. **(A)** Cntnap2 (green) colocalizes with a subset of $Sox10^+$ cells (magenta) (arrowheads). Enteric neurons labeled with HuC/D (blue). $Cntnap2^+$ neurons are indicated with asterisks. **(B)** Cntnap2 (green) colocalizes with a subset of $5\text{-}HT^+$ cells (magenta) in the epithelium. **(C, D)** $Cntnap2^+$ (green) cell bodies and projections, as well as $Sox10^+$ cells (magenta), are visible using 0.1% PBT (C). $Cntnap2^+$ cell bodies are more pronounced with 0.01% PBT, but we no longer detect Sox10+ labeling (D). Scale bar, (A, B) 10 µm, (C, D) 50 µm.
